# Improved Mitochondrial Function with Diet-Induced Increase in Either Docosahexaenoic Acid or Arachidonic Acid in Membrane Phospholipids

**DOI:** 10.1371/journal.pone.0034402

**Published:** 2012-03-30

**Authors:** Ramzi J. Khairallah, Junhwan Kim, Karen M. O'Shea, Kelly A. O'Connell, Bethany H. Brown, Tatiana Galvao, Caroline Daneault, Christine Des Rosiers, Brian M. Polster, Charles L. Hoppel, William C. Stanley

**Affiliations:** 1 Division of Cardiology, Department of Medicine, University of Maryland, Baltimore, Maryland, United States of America; 2 Department of Medicine and Pharmacology, Case Western Reserve University, Cleveland, Ohio, United States of America; 3 Department of Nutrition and Montreal Heart Institute, Université de Montréal, Montreal, Quebecc, Canada; 4 Department of Anesthesiology, and Shock, Trauma, and the Anesthesiology Research (STAR) Center, University of Maryland, Baltimore, Maryland, United States of America; University of Las Palmas de Gran Canaria, Spain

## Abstract

Mitochondria can depolarize and trigger cell death through the opening of the mitochondrial permeability transition pore (MPTP). We recently showed that an increase in the long chain n3 polyunsaturated fatty acids (PUFA) docosahexaenoic acid (DHA; 22:6n3) and depletion of the n6 PUFA arachidonic acid (ARA; 20:4n6) in mitochondrial membranes is associated with a greater Ca^2+^ load required to induce MPTP opening. Here we manipulated mitochondrial phospholipid composition by supplementing the diet with DHA, ARA or combined DHA+ARA in rats for 10 weeks. There were no effects on cardiac function, or respiration of isolated mitochondria. Analysis of mitochondrial phospholipids showed DHA supplementation increased DHA and displaced ARA in mitochondrial membranes, while supplementation with ARA or DHA+ARA increased ARA and depleted linoleic acid (18:2n6). Phospholipid analysis revealed a similar pattern, particularly in cardiolipin. Tetralinoleoyl cardiolipin was depleted by 80% with ARA or DHA+ARA supplementation, with linoleic acid side chains replaced by ARA. Both the DHA and ARA groups had delayed Ca^2+^-induced MPTP opening, but the DHA+ARA group was similar to the control diet. In conclusion, alterations in mitochondria membrane phospholipid fatty acid composition caused by dietary DHA or ARA was associated with a greater cumulative Ca^2+^ load required to induced MPTP opening. Further, high levels of tetralinoleoyl cardiolipin were not essential for normal mitochondrial function if replaced with very-long chain n3 or n6 PUFAs.

## Introduction

Cardiac muscle is densely packed with mitochondria, which are essential to support the high rate of ATP generation needed for contractile function. Mitochondria also are important for cell survival, as under conditions of stress they can depolarize and trigger cell death through the opening of the mitochondrial permeability transition pore (MPTP). The structure and regulation of the MPTP is not well understood [1], [2]. We recently found that increased long chain n3 polyunsaturated fatty acid (PUFA) and depletion of n6 PUFA in mitochondrial membrane phospholipids induced by high intake of docosahexaenoic acid (DHA; 20:6n3) were associated with resistance to Ca^2+^-induced MPTP opening [3]–[5]. Long chain PUFAs, specifically DHA and arachidonic acid (ARA; 20:4n6), are structurally distinguished from less unsaturated fatty acids such as oleic acid (18:1n9) or linoleic acid (18:2n6) by repeating double bonds that produces a highly flexible chain and a more fluid membrane [6]. DHA is the most unsaturated PUFA commonly found in mammals, followed by eicosapentaenoic acid (EPA; 20:5n3) and ARA. DHA supplementation has shown promise as a means to prevent and treat heart failure, which may be partially mediated by improvements in mitochondrial function [7]–[9].

ARA is depleted by the increase in membrane phospholipid DHA content induced by dietary DHA supplementation. This could be beneficial, as ARA is a precursor of inflammatory eicosanoids, and can also trigger MPTP opening when released from cell membranes by phospholipase A [10], [11]. Thus the greater Ca^2+^ load required to induce MPTP opening with DHA supplementation may occur secondary to lowering ARA in membrane phospholipids. If true, then an increase in ARA in mitochondrial membrane phospholipids above normal levels is predicted to increase MPTP opening.

Like other cardiac membranes, mitochondrial phospholipids are mainly comprised of phosphotidylethanolamine (PE) and phosphotidylcholine (PC), however they are unique in that they contain the tetra-acyl phospholipid cardiolipin (CL). CL comprises 15–20% of the mass of total mitochondrial phospholipid [12]. Depletion of CL, as seen in Barth syndrome patients who have an inherited defect in CL synthesis, results in severe mitochondrial dysfunction and cardiomyopathy [13]. Linoleic acid is the main fatty acyl moiety in CL, with 60–80% of CL being tetralinoleoyl CL (L_4_CL) in cardiac mitochondria in humans, dogs and rats [12], [14], [15]. It has been proposed that high levels of L_4_CL are essential for optimal mitochondrial function in the heart [12]. Evidence to the contrary comes from the mouse heart, where L_4_CL comprises only 22% of the total CL [14], and is replaced by CL species that contain DHA (53% of total CL) [14], [15]. A decrease in the total CL in cardiac mitochondria and less L_4_CL has been observed in acquired cardiac pathologies such as hypertension-induced hypertrophy and heart failure in rodents [12], but not dogs [16]. Importantly, there is also evidence that high intake of fish oil rich in DHA can increase CL in heart mitochondria [3], [17], [18], though this is not a consistent finding [5]. The effect of ARA intake on CL has not been reported, but presumably would increase ARA incorporation into all mitochondrial phospholipids and could alter mitochondrial function.

In the present investigation we used pharmacological levels of DHA and ARA (2.5% of energy intake [19], [20]), well above those consumed in food by humans, to manipulated cardiac mitochondrial phospholipid composition, and assessed the subsequent effects on respiratory function and susceptibility to MPTP opening in isolated cardiac mitochondria. We hypothesized that replacing linoleic acid with either DHA or ARA in mitochondrial membrane phospholipids would not adversely affect mitochondria respiratory function in the absence of stress, but that ARA would increase susceptibility to Ca^2+^-induced MPTP opening. We further hypothesized that dietary ARA supplementation would dramatically increase ARA in mitochondrial phospholipids, and specifically decrease L_4_CL and increase incorporation of ARA side chains of CL. Rats were fed diets supplemented with DHA, ARA or combined DHA+ARA at physiologically relevant doses. Cardiac contractile function was evaluated, and cardiac mitochondria were analyzed for susceptibility to MPTP opening, total phospholipid fatty acid composition, and individual molecular species within each phospholipid class by mass spectrometry.

## Results

### Morphometric data

All groups had similar weight gain and food consumption, and there were no significant differences in body, heart or liver mass between dietary treatments (Table 1). There was no effect of diet on LV dimensions as measured by echocardiography (Table 1).

**Table 1:** Body mass, organ mass, and LV dimensions.

	Control	DHA	ARA	DHA+ARA
**Group size:**	15	14	15	15
**Terminal Body Mass (g)**	501±11	500±6	502±9	505±9
**LV Mass (g)**	0.895±0.021	0.917±0.017	0.902±0.020	0.938±0.024
**LV/Tibia length (g/cm)**	0.22±0.01	0.23±0.00	0.22±0.01	0. 23±0.01
**RV Mass (g)**	0.262±0.011	0.267±0.004	0.268±0.004	0.269±0.008
**Biatrial Mass (g)**	0.083±0.003	0.091±0.006	0.092±0.005	0.083±0.003
**Liver Mass (g)**	13.6±0.6	12.5±0.3	13.3±0.4	14.1±0.5
**Mitochondrial Yield (mg mito protein/g wet wt)**	14.57±0.69	14.17±0.64	14.42±0.86	15.52±1.00
**End-systolic diameter (mm)**	0.77±0.01	0.76±0.02	0.77±0.02	0.81±0.02
**End-diastolic diameter (mm)**	0.43±0.02	0.43±0.01	0.43±0.02	0.48±0.02
**Fractional shortening**	0.45±0.02	0.43±0.02	0.44±0.02	0.41±0.05
**Ejection fraction (%)**	83.0±1.4	81.3±1.5	81.9±1.8	79.2±1.4
**Malonaldehydes+Hydroxyalkenals (nmols/mg protein)**	0.56±0.04	0.58±0.04	0.53±0.03	0.68±0.04* †

Data are the mean ± SEM. LV, left ventricle. RV, right ventricle.

* p<0.05 vs CTRL.

† p<0.05 vs ARA.

### Mitochondrial Phospholipid Composition

**Figure 1:**
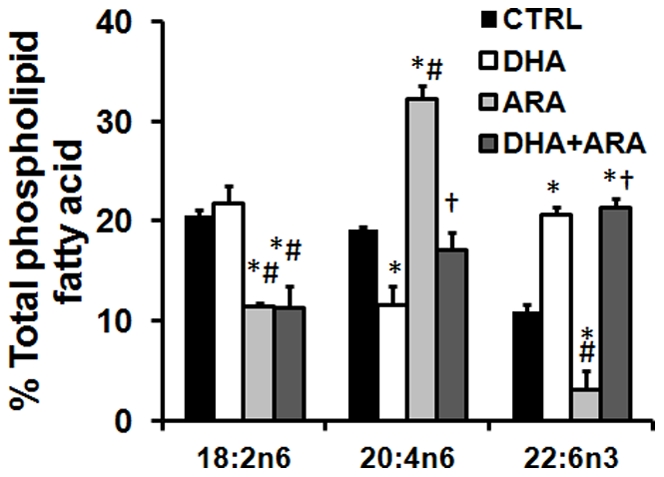
Cardiac mitochondrial phospholipid fatty acid composition. Values are expressed as percentage of total fatty acids. Data are mean±SEM. CTRL, n = 7. DHA, n = 7. ARA, n = 5. DHA+ARA, n = 6.

The fatty acid composition of total mitochondrial phospholipids was dramatically altered by all 3 dietary interventions (Table 2, Figure 1). Both diets containing DHA raised mitochondrial phospholipid DHA content approximately 2-fold, while ARA supplementation led to a 70% reduction in DHA. Conversely, ARA supplementation increased membrane ARA by ∼50% while DHA supplementation decreased ARA by ∼50%. Combined DHA+ARA supplementation maintained ARA content at levels seen in the control diet group. Membrane EPA was increased from undetectable levels to approximately 1% by both the DHA and ARA diets, but was undetectable with the combined DHA+ARA diet. Oleate was only slightly affected by the different diets (Table 2). Most notably, the double bond index, a measure of membrane unsaturation, and the n3/n6 ratio were both dramatically decreased with ARA supplementation and preserved with the DHA+ARA diet.

**Table 2:** Mitochondrial phospholipid fatty acid composition expressed as molar percent of total phospholipid fatty acid.

Fatty Acid	CTRL	DHA	ARA	DHA+ARA
**Palimate (C16:0)**	16.0±0.4	17.7±1.0	14.8±0.6#	16.8±0.7
**Stearate (C18:0)**	25.4±0.7	21.5±2.2	27.2±4.1	25.7±0.6
**Oleate (C18:1n9)**	4.1±0.3	3.3±0.3	3.6±0.	2.7±0.1*
**Vaccenic acid(C18:1n7)**	4.3±0.1	3.3±0.3*	4.1±0.2#	3.6±0.2
**Linoleic acid (C18:2n6)**	20.5±0.6	21.7±1.8	11.4±0.5#	11.4±2.1#
**Arachidonic acid (20:4n6)**	19.1±0.3	11.5±2.0*	32.2±1.3* #	17.2±1.8
**Eicosapentaenoic Acid (20:5n3)**	BQL	1.5±0.3*	0.9±0.4*	BQL
**Docosahexanoic acid (22:6n3)**	10.9±0.7	20.7±0.7*	3.1±2.0* #	21.4±0.9* †
**Double Bond Index**	191±4.0	221±8	183±15	227±5* †
**n3/n6 ratio**	0.27±0.01	0.73±0.04	0.15±0.03#	0.73±0.04* †

Data are means±SEM. CTRL, n = 7. DHA, n = 7. ARA, n = 5. DHA+ARA, n = 6.

* p<0.05 vs CTRL.

# p<0.05 vs DHA,

† p<0.05 vs ARA; BQL, Below Quantifiable Limit.

**Figure 2:**
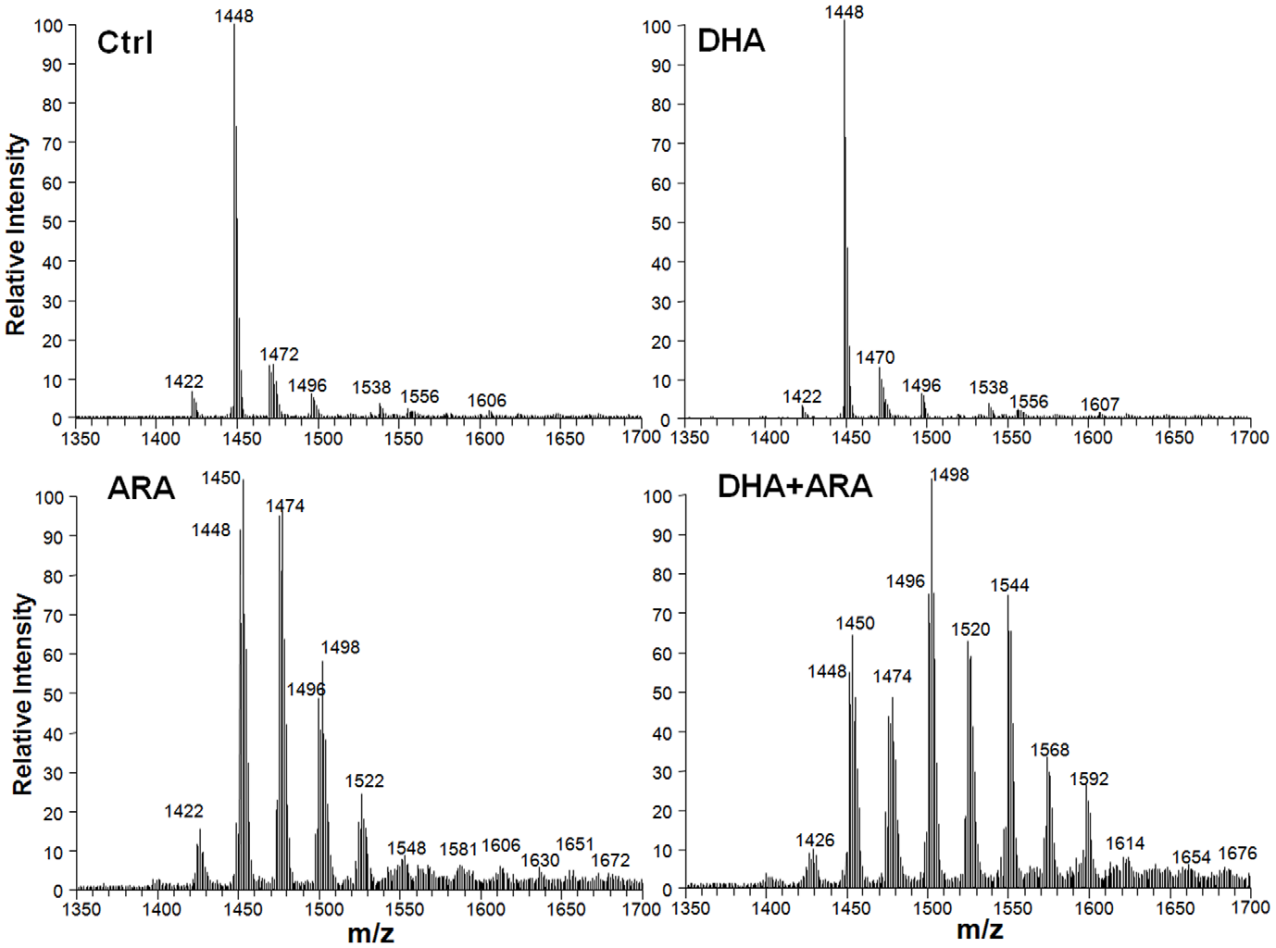
Representative mass spectra of cardiac mitochondrial CL species.

Phospholipid classes were not changed by diet to any significant extent, except for a decrease in CL and mono-lyso-CL (MLCL) with ARA and DHA+ARA supplementation (Table 3). There was also a small increase in PC with ARA supplementation. In contrast, analysis of side chain composition within each phospholipid class revealed some dramatic diet-induced changes in fatty acyl groups (Table S1, Figure 2, S1,S2,S3,S4,S5). Dietary supplementation with DHA increased DHA in PE, PI and PC, as assessed by mass spectrometry. The increase in DHA was determined by the increase in peak intensity at molecular masses that corresponded to the calculated theoretical mass based on probable side chains (Table S1). Similarly, ARA was increased by the ARA and DHA+ARA diets in PE, PG, PC, CL and MLCL.

**Table 3:** Composition of cardiac mitochondrial membrane by phospholipid class.

Phospholipid Class (mol% of total)	CTRL	DHA	ARA	DHA+ARA
**CL**	8.5±0.2	8.5±0.2	7.0±0.1* #	7.2±0.1* #
**MLCL**	0.5±0.0	0.5±0.0	1.0±0.1* #	0.7±0.0* # ^†^
**PE**	47.7±0.5	48.2±0.6	46.1±0.5#	46.9±0.5
**PI**	3.9±0.1	3.7±0.1	3.8±0.1	4.1±0.1
**PG**	0.6±0.0	0.6±0.1	0.7±0.1#	0.6±0.0
**PC**	38.7±0.5	38.6±0.6	41.5±0.5* #	40.5±0.6

Data are means±SEM. CTRL, n = 13. DHA, n = 12. ARA, n = 14. DHA+ARA, n = 14. CL, cardiolipin. MLCL, monolysocardiolipin. PE, phosphotidylethanolamine. PI, phosphotidylinositol. PG, phosphotidylglycine. PC, phosphotidylcholine.

* p<0.05 vs CTRL.

# p<0.05 vs DHA.

**Figure 3:**
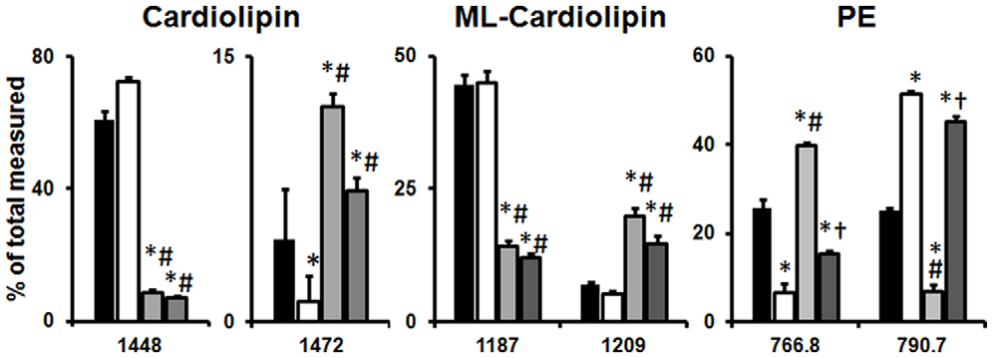
Cardiac mitochondrial phospholipid species composition. Values are expressed as percentage of total measured. Data are mean±SEM. CTRL, n = 14. DHA, n = 13. ARA, n = 13. DHA+ARA, n = 14. *p<0.05 vs CTRL. #p<0.05 vs DHA. †p<0.05 vs ARA.

There was clear interaction between dietary DHA and ARA in their effect on phospholipid fatty acid composition, with DHA alone depleting ARA from CL, PE and PC (Figures 2 and 3, Table S1). Conversely, dietary ARA depleted DHA from PE and PC. Interestingly, the combination of DHA and ARA restored both DHA and ARA in PE and PC. Further, it gave a profile that was largely similar to the diet with ARA alone. This is illustrated in the mass spectrum (Figure 2 for CL, and Figures S1,S2,S3,S4,S5 for other phospholipids), which shows that treatment with ARA and DHA+ARA resulted in a profound shift in the profile, with an increase in peak intensities for higher molecular weight species. On the other hand, DHA had far less of an effect on the profile. As noted above, treatment with ARA or DHA+ARA, but not DHA alone, depleted linoleic acid content from total phospholipids by ∼50% (Figure 1), and the analysis of individual phospholipid species revealed that this occurred in CL, MLCL, PE PG and PC (Figure 3, Table S1).

### Lipid Peroxidation

DHA+ARA supplementation significantly increased myocardial content of malonaldehydes and hydroxyalkenals compared to the control and ARA groups (p<0.05), and showed a strong trend toward being higher than the DHA group (p = 0.071).

### Urine Thromboxane B_2_

TBX B_2_ is a metabolite of pro-inflammatory thromboxane A_2_, and is excreted by the kidneys. Both diets containing ARA increased TBX B_2_ excretion significantly by two- to three-fold compared to CTRL and DHA diets (29.0±2.9 and 22.8±2.6 pg/µmol creatine for CTRL and DHA groups, vs. 63.3±6.7 and 50.11±4.8 for ARA and DHA+ARA groups, respectively; p<0.05)

### Mitochondrial Respiration

There was no effect of diet on State 3 or State 4 respiration, or on the respiratory control ratio (RCR) with any of the substrates used (Table 4).

**Table 4:** Mitochondrial Respiration.

	Control	DHA	ARA	DHA+ARA
**Pyruvate+Malate**				
State 3	225±17	239±22	245±21	236±19
State 4 (−oligomycin)	51.6±2.0	51.3±3.1	47.3±2.6	49.7±3.7
State 4 (+oligomycin)	36.8±4.1	44.8±4.3	33.7±3.6	41.1±4.3
RCR (−oligomycin)	4.5±0.4	4.7±0.4	4.8±0.2	5.2±0.4
RCR (+oligomycin)	6.8±0.8	5.8±0.6	8.1±0.9	6.2±0.6
**Palmityl-CoA+Carnitine+Malate**				
State 3	221±16	203±14	200±14	196±13
State 4 (−oligomycin)	45.6±2.9	53.4±5.0	46.0±2.6	46.6±2.9
State 4 (+oligomycin)	40.9±3.8	41.2±4.7	36.4±2.8	42.9±4.3
RCR (−oligomycin)	5.0±0.3	4.1±0.4	4.3±0.3	4.5±0.4
RCR (+oligomycin)	5.7±0.4	6.4±1.7	5.8±0.5	5.1±0.6
**Succinate+Rotenone**				
State 3	392±16	398±19	360±22	393±25
State 4 (−oligomycin)	122.7±3.6	120.1±7.3	111.6±4.7	108.2±6.9
State 4 (+oligomycin)	107.8±4.9	123.9±7.8	100.0±8.3	123.6±7.0
RCR (−oligomycin)	3.2±0.1	3.4±0.2	3.7±0.2	3.2±0.2
RCR (+oligomycin)	3.7±0.1	3.3±0.1	3.8±0.3	3.2±0.1

Data are the mean ± SEM. CTRL, n = 14. DHA, n = 13. ARA, n = 13. DHA+ARA, n = 14. All Rates are expressed in ng atoms O·mg^−1^·min^−1^. The RCR, defined as the ratio of State 3 to State 4 respiration rate, was calculated from the State 4 rate with oligomycin.

### Ca^2+^ Retention Capacity

**Figure 4:**
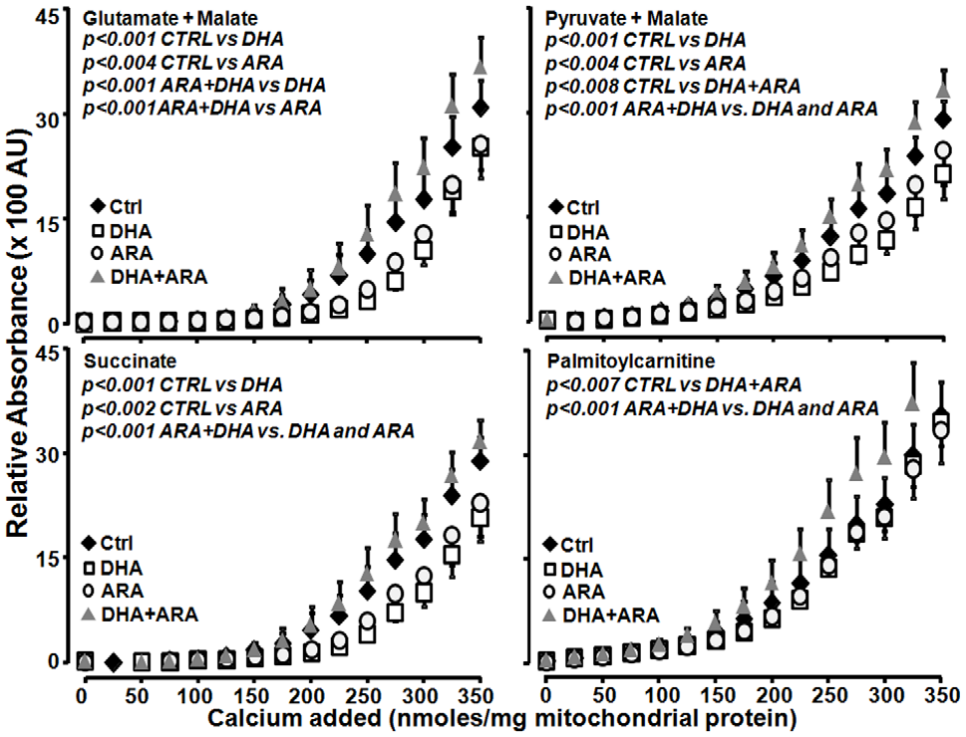
Effect of diet on Ca^2+^ retention capacity in the presence of different respiratory substrates. Data are mean±SEM. CTRL, n = 9. DHA, n = 9. ARA, n = 9. DHA+ARA, n = 10.

Mitochondria from rats supplemented with DHA or ARA alone had significantly enhanced Ca^2+^ retention capacity compared to CTRL animals, as reflected by significantly lower extramitochondrial [Ca^2+^] for a given cumulative Ca^2+^ load with all substrates except palmitoylcarnitine+malate (Figure 4). Surprising, dietary supplementation with the combination of DHA+ARA resulted in greater sensitivity to Ca^2+^ MPTP opening, as reflected in a higher extramitochondrial [Ca^2+^] with all substrates when compared to either CTRL, DHA or ARA with pyruvate+malate as a substrate (Figure 4).

**Figure 5:**
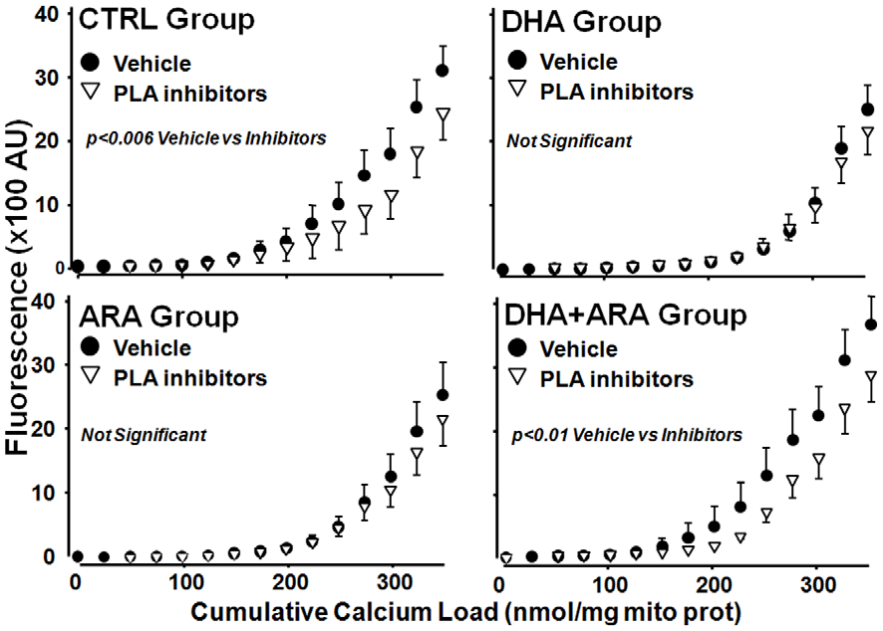
Effect of phospholipase A_2_ inhibition on the Ca^2+^ retention capacity. Data are mean ±SEM. CTRL, n = 9. DHA, n = 9. ARA, n = 9. DHA+ARA, n = 10. CTRL vehicle vs CTRL inhibitors p<0.006. DHA+ARA vehicle vs DHA+ARA inhibitors p<0.01.

To assess the possible role of Ca^2+^-dependent and –independent phospholipase A_2_ in Ca^2+^ induced MPTP opening, we assessed the effects of addition of aristolochic acid and bromoenolactone, inhibitors of phospholipase A_2_, on the relationship between cumulated Ca^2+^ load and extramitochondrial [Ca^2+^]. This shifted the relationship to the right in mitochondrial from the CTRL and DHA+ARA groups, but not in the DHA or ARA groups (Figure 5).

### Mitochondrial Swelling

**Figure 6:**
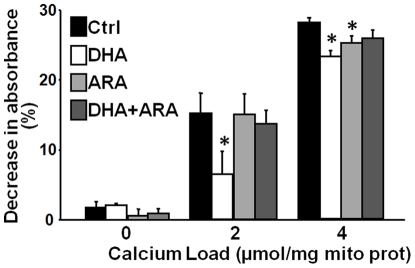
Effect of diet on mitochondrial Ca^2+^ induced swelling. Average decrease in absorbance 900 seconds after Ca^2+^ addition. Data are mean ± SEM. CTRL, n = 9. DHA, n = 8. ARA, n = 9. DHA+ARA, n = 9. * p<0.05 vs CTRL.

The decrease in absorbance at 520 nm following the addition of Ca^2+^ to isolated mitochondria was used as a measure of MPTP opening. DHA supplementation attenuated the in response to 2 µmols Ca^2+^/mg mitochondrial protein compared to CTRL, ARA and DHA+ARA groups (Figure 6). Both DHA and ARA supplementation attenuated the decrease in absorbance in response to 4 µM µmols Ca^2+^/mg mitochondrial protein compared to CTRL.

## Discussion

Here we present the novel observation that a large increase in either DHA or ARA in mitochondrial membrane phospholipids is associated with a significant increase in the mitochondrial capacity for Ca^2+^ retention, an index of MPTP opening. Further, we showed that L_4_CL is not essential for normal mitochondrial function if replaced with very-long chain n3 or n6 PUFA. On the other hand, the extreme increase in the sum of ARA and DHA accompanied by depletion of linoleic acid that occurred with the DHA+ARA diet was associated with increased susceptibility to MPTP opening, and suggests that the combination of elevated DHA and ARA with low linoleic acid in membrane phospholipids is detrimental.

Our data do not support our initial hypothesis that the beneficial effect of DHA on MPTP opening is due to decreased membrane phospholipid ARA content. ARA has been identified as an MPTP inducer [21] and increased free ARA in cells can result in necrosis or apoptosis [10], [22], [23]. For example, inhibition of mitochondrial Ca^2+^-independent phospholipase A_2_γ, which is predicted to lower free ARA, reduced Ca^2+^-induced MPTP opening, mitochondrial phospholipid loss and reduced infarct size following I/R [23]. Similarly, Penzo and colleagues demonstrated that ARA released by Ca^2+^-dependent cytosolic phospholipase A_2_ activation triggers MPTP opening and cell death [11]. We found that dual inhibition of Ca^2+^-dependent and independent phospholipase A_2_ enzymes with a combination of aristolochic acid and bromoenolactone, respectively, resulted in delayed Ca^2+^-induced MPTP in the mitochondria of control-fed rats. Interestingly, it also provided a protective effect in the DHA+ARA group and altered the calcium sensitivity back to control levels. Both control and DHA+ARA groups, with phospholipase inhibition, are not significantly different from either DHA or ARA groups. This supports the conclusions made by previous studies in favor of a role for ARA release by phospholipases in inducing MPTP opening. However, we also saw that ARA supplementation does not induce MPTP opening as this hypothesis would predict, but rather delays it with no added benefit of phospholipase inhibition. Similarly, in the DHA group phospholipase inhibition did not provide additional resistance in MPTP opening. Thus, it is unlikely that modulation of free ARA explains the downward shift in the relationship between cumulative Ca^2+^ load and extramitochondrial [Ca^2+^] induced MPTP opening seen with ARA or DHA supplementation.

Although ARA supplementation provided a protective effect on MPTP opening, it also increased thromboxane production. This increase in inflammatory mediators also was seen in the combined DHA+ARA diet. The potential for ARA to modulate inflammation has been described by others [24]. Prostaglandin E_2_ synthesis can be increased by dietary supplementation with ARA, and this is modulated by other saturated fatty acids [25]–[27]. However, given the potential for ARA to increase inflammation, it is necessary to reconsider its use as a dietary supplement to prevent MPTP, especially in diseases that have an inflammatory component to their pathology, such as heart failure.

The combined DHA+ARA diet did not provide the protective effect of the diets containing DHA or ARA alone, and was detrimental to MPTP opening and increased inflammatory markers. The very high dose of PUFA provided by this diet increases membrane unsaturation to very high levels. Taking into account the high susceptibility of the highly unsaturated fatty acids to undergo peroxidation, and their proximity in the mitochondria to an ample source of reactive oxygen species, the DHA+ARA diet may increase MPTP susceptibility due to increased lipid peroxidation. This is further supported by the increase in malonaldehydes and hydroxyalkenals seen in the combined diet. Thus, the optimal dose of PUFAs might be below the threshold where increased lipid peroxidation outweighs the benefits provided by increasing membrane unsaturation. A less likely possibility is that there is negative interaction between DHA and ARA that sensitizes the MPTP. To fully exclude this type of effect, future studies should compare DHA alone and ARA alone to DHA+ARA at an equivalent dose of total PUFA.

We did not observe any differences in mitochondrial respiration with DHA supplementation with any of the substrates tested. We had previously shown that DHA supplementation decreases state 4 respiration with pyruvate+malate as substrates [3]. A subsequent study failed to replicate this finding [4], and again, we saw no difference in state 4 respiration in this study. The larger sample size used here allows for more confidence in concluding that DHA does not affect non-phosphorylating respiration in the healthy mitochondria compared to the CTRL diet. This, however, may not apply to mitochondria that have been stressed. Given DHA's protective effect in heart failure [7]–[9], we expect that DHA supplementation would ameliorate respiratory defects associated with heart disease.

There are limitations to the present investigation that need to be addressed. First, we assess only subsarcolemmal mitochondria, and not the whole population of cardiac mitochondria that includes the subpopulation of cardiac mitochondrial found amongst the fibrils (“interfibrillar mitochondria”). Isolation of total cardiac mitochondria requires incubation with a protease (trypsin) to disrupt the fibrils and release interfibrillar mitochondria, however we did not want to risk potential damage to mitochondrial proteins by this procedure. The effects protease digestion on mitochondrial Ca^2+^ uptake or MPTP opening is not known, thus we did not wish to introduce this potential confounding variable. We have previously found that there are no differences in membrane phospholipid fatty acid composition between interfibrillar and subsarcolemmal cardiac mitochondria in rats fed a standard low n-3 PUFA lab chow, or in the changes in mitochondrial membrane phospholipids caused by long term dietary supplementation with DHA or eicosapentaenoic acid (EPA) [4], [5]. This suggests that the changes we observed in subsarcolemmal mitochondria in the present study would very likely be duplicated in interfibrillar mitochondria. A second important limitation is that lack of measurement of the initial mitochondrial [Ca^2+^], as dietary supplementation with DHA or ARA could affect the residual Ca^2+^ in the mitochondrial matrix following isolation. Decreased initial matrix [Ca^2+^] could increase mitochondrial Ca^2+^ uptake capacity and give the impression of delayed PTP opening in response to successive Ca^2+^ additions. Our main conclusion regarding mitochondrial Ca^2+^ retention is that dietary supplementation with docosahexaenoic acid or arachidonic acid are associated with a greater exogenous Ca^2+^ load required to induce MPTP opening. We know of no rationale to suggest there would be differences in the initial [Ca^2+^] among treatment groups, nevertheless there may be differences that could affect the capacity for Ca^2+^ retention. Clearly future studies should assess this variable. Lastly, we did not assess the effects of cyclosporin A on Ca^2+^-induced MPTP opening in the present investigation. In a previous study we found that acute *ex vivo* treatment with cyclosporine A delayed Ca^2+^-induced MPTP opening, on rats fed a normal diet or with supplementation with DHA+EPA or EPA, but not DHA alone [4], [5]. Future studies should compare the effects of ARA and DHA supplementation on the delay in MPTP opening caused by cyclosporine A.

In summary, dietary supplementation with DHA or ARA caused dramatic alterations in mitochondria membrane phospholipid fatty acid composition, and delayed Ca^2+^-induced MPTP opening to approximately the same extent when compared to the standard control diet. This suggests that the effect of DHA on MPTP is most likely due to membrane unsaturation rather than depletion of ARA. Furthermore, DHA+ARA supplementation showed no added benefit, but rather sensitized MPTP to opening, suggesting that a very large dose of long chain PUFA can be detrimental. Finally, we found that high levels of L_4_CL were not essential for normal mitochondrial function if replaced by very-long chain n3 or n6 PUFAs.

## Methods

### Experimental Design

The animal protocol was conducted according to the Guideline for the Care and Use of Laboratory Animals (NIH publication 85-23) and was approved by the University of Maryland School of Medicine Institutional Animal Care and Use Committee (Protocol number 1009011). In accordance with this document, every effort was made to prevent pain to the animal, and to aleviate pain with anesthetic when it could potentially occur. Investigators were blinded to treatment when measurements were performed. The animals were maintained on a reverse 12-h light–dark cycle and all procedures were performed in the fed state between 3 and 6 h from the start of the dark phase. Male Wistar rats weighing 190–200 g were fed a standard low fat diet (CTRL) or a modified standard diet containing DHA or ARA at 2.5% of total caloric intake, which corresponds to a human intake of approximately 5.5 g/day (calculated assuming an energy intake of 2000 kcal/day and 9 kcal/g of fat) or a combined DHA+ARA diet at 5% of total caloric intake. After 10 weeks of dietary treatment, rats were anesthetized with isoflurane, urine was collected from the bladder, and hearts were harvested for biochemical analysis and mitochondrial isolation. Urine was analyzed for creatinine and thromboxane metabolites. Cardiac mitochondria were analyzed for respiration, Ca^2+^ retention capacity, Ca^2+^ induced swelling and membrane phospholipid composition as described below.

### Diets

All diets were custom-manufactured (Research Diets Inc., New Brunswick, NJ), and had 66% of total energy from carbohydrate (54% of total energy from cornstarch and 12% from maltodextrin), 20% protein (casein supplemented with l-cystine) and 14% energy from fat (see Table 5 for fatty acid composition). In the CTRL diet, the fat was made up of 71.5% cocoa butter, 17.1% soybean oil, 7.2% palm oil, 2.8% safflower oil and 1.4% linseed oil. The DHA diet contained 5.75% of total energy from DHASCO (Martek Inc, Columbia, MD, USA) that was comprised of 43.6% DHA by, in place of part of the cocoa butter. The DHASCO oil was free from ARA. The ARA diet had 6.25% of energy from ARASCO (Martek Inc, Columbia, MD, USA) that was comprised of 40.1% ARA by mass partially replaced cocoa. The ARASCO oil was free from DHA. The DHA+ARA diet had both the DHA and ARA enriched oils added at 5.75 and 6.25% of energy respectively. DHASCO and ARASCO oils were purified from algae and contained ascorbyl palmitate (250 ppm) and tocopherols (250 ppm) to prevent lipid oxidation, which was less than 0.5 meq/kg at the time of manufacture of the diet. All diets were supplemented with the same amount of vitamins (Vitamin Mix V10001, 10 g/kg), minerals (Mineral Mix S10026, 10 g/kg), cellulose (50 g/kg) and choline (2 g/kg). It is important to note that the levels of DHA and ARA used in this study are equivalent to ∼5.5 g/day in humans (calculated assuming an energy intake of 2000 kcals/day). Tthus the dose of DHA and ARA use in this study are pharmacological, and are far above what is typically consumed in food (∼0.0.5 to ∼0.7 g/day of each) [19], [20].

**Table 5:** Fatty acid compositions of the rodent diets expressed as the molar percent of total fatty acids in the diet.

Fatty Acid		Diet		
	CRTL	DHA	ARA	DHA+ARA
**C12:0**	3.4	1.5	3.4	1.5
**C14:0**	1.1	3.6	1.6	4.1
**C16:0**	21.7	14.6	16.2	9.5
**C16:1**	0.2	1.4	0.1	1.4
**C18:0**	25.8	14.2	16.1	5.3
**C18:1n-9**	30.3	28.8	23.4	21.9
**C18:2n6**	13.9	14.7	15.2	14.1
**C18:3n3**	2.2	2.2	2.3	2.4
**C20:4n6**	-	-	18.3	18.4
**C20:5n3**	-	0.3	-	0.3
**C22:6n3**	-	17.8	-	18.3
**Total saturated**	52	34	37	20
**Total mono-sat**	30	30	23	23
**Total n3**	2.2	20	2.3	21
**Total n6**	14	15	33	32
**n6/n3**	6.3	0.7	15	1.5
**PUFA/saturated**	0.3	1.0	1.3	2.6

All diets had 14% of total energy from fat, 20% from protein and 66% from carbohydrates.

### Echocardiography

Since dietary lipids can effect cardiac chamber size and contractile function, we assessed LV dimensions in anesthetized rats using a high resolution small animal imaging system (model Vevo 770, with transducer model RMV 716, VisualSonics Inc., Toronto, Canada), as previously described in detail [5]. Briefly, rats were anesthetized with isoflurane by mask, placed supine on a heated platform, and 2-dimensional cine loops and guided M-mode frames were acquired from the short and long axis. Measurements were made off line using software resident on the system, with the investigator blind to the treatment.

### Metabolic and Biochemical Parameters

Lipid peroxidation was measured in whole tissue homogenate using a commercially available kit reacting to malonaldehydes and 4-hydroxyalkenals (Oxford Biomedical Research, MI, USA). Urine creatinine and thromboxane B_2_ were measured using commercially available kits (Cayman Chemicals, MI, USA)

### Mitochondrial Preparation

Subsarcolemmal mitochondria were isolated as previously described [3], [26]. LV tissue (approximately 500 mg) was thoroughly rinsed and cut immediately following excision of the heart and placed in Ca^2+^ free ice-cold modified Chappel-Perry buffer (100 mM KCl, 50 mM MOPS, 5 mM MgSO_4_, 1 mM Na_2_ATP, 1 mM EGTA, 2 mg/ml BSA). LV tissue was dissected, rinsed again, blotted dry, then minced in ice-cold buffer. Prior to homogenization, the buffer was replaced twice to remove blood contamination. The tissue was homogenized in 1∶10 (wt/vol) Chappel-Perry buffer, and subsarcolemmal mitochondria were then isolated and purified by differential centrifugation. Mitochondrial protein concentration was measured by the Lowry method using bovine serum albumin (BSA) as a standard.

### Membrane Lipid Composition

Cardiac phospholipid fatty acid composition was assessed in isolated cardiac mitochondria homogenates by gas chromatography coupled with mass spectrometry according to a modification of the transesterification method as previously described [28]. Individual phospholipid species were quantified after extraction using the method of Christiansen in the presence of internal standard [29]. The resultant extract was subjected to silica gel chromatography to harvest phospholipids [30]. The phospholipid classes were separated on a normal-phase HPLC coupled to a LCQ Deca (Thermo Scientific) ion trap mass spectrometer. Quantitation was performed by interpolation to standard curves for each class of phospholipids. (Kim J. & Hoppel C.L., manuscript submitted). Mass spectrometric data was adjusted for natural abundance using predicted molecular composition from the RCM Lipid Calculator (http://pharmacology.ucdenver.edu/lipidcalc/Default.aspx) and the GENEBIO SmileMS Low Precision Isotope Distribution Calculator (http://research.smilems.com/molecule-tk/moleculeUtils/isotoperatio).

### Mitochondrial respiration

Mitochondrial oxygen consumption was measured using a Clark-type oxygen electrode (Qubit Systems, Ontario, Canada). Mitochondria (0.25 mg protein) were suspended in respiration buffer (0.5 ml; 100 mM KCl, 50 mM MOPS, 5 mM KH_2_PO_4_ 1 mM EGTA, and 0.5 mg fatty acid-free BSA) at pH 7.4 and 37°C [31]. State 3 (ADP-stimulated) and state 4 (non-phosphorylating) respiration were measured with 3 different sets of substrates: pyruvate+malate (10 and 5 mM, respectively) and palmityl-CoA+carnitine+malate (40 µM, 5 mM and 5 mM, respectively) to assess respiration through complex I–IV, and succinate+rotenone (10 mM and 7.5 µM, respectively) to assess respiration through complex II–IV of the ETC exclusively. Oligomycin was also used to inhibit mitochondrial ATP synthase and assess state 4 respiration.

### Mitochondrial Ca^2+^ Retention Capacity

Mitochondrial Ca^2+^ retention was assessed using a 96-well fluorescence plate reader (FLUOstar Optima, BMG Labtech, Germany) as previously described [3]. 25 µg of mitochondria per well were resuspended in 200 µL of the same buffer used above, but with varying substrates and inhibitors; either glutamate+malate (10 and 5 mM, respectively), pyruvate+malate (10 and 5 mM, respectively), palmitoylcarnitine+malate (40 µM and 5 mM, respectively) or succinate (10 mM) with rotenone (7.5 µM), and glutamate+malate with phospholipase A_2_ inhibitors bromoenolactone (5 µM) and aristolochic acid (5 µM). Extramitochondrial Ca^2+^ was monitored using 1 µM Calcium Green 5N and fluorescence was measured at 485 nm and 538 nm for excitation and emission wavelengths respectively. Automated additions of 25 nmoles Ca^2+^/mg mitochondrial protein were performed at regular 7 minute intervals and fluorescence measured every 17 seconds for 160 min at 37°C.

### Ca^2+^-Induced Mitochondrial Swelling

Light scattering, an index of Ca^2+^-induced mitochondrial swelling, was monitored using a 96 well spectrophotometric plate reader (SpectraMax, Molecular Devices, USA) [3]. Briefly, 25 µg of mitochondria were resuspended in 200 µl of the same buffer as used for the Ca^2+^ retention capacity assay. Baseline absorbance at 540 nm was read at 7 second intervals for 2 min, then either 50 or 100 nmoles of Ca^2+^ was rapidly added to the wells and the absorbance was read for 15 min at 37°C.

### Statistical Analyses

Mean values are presented ± SEM, and the level of significance was set at p<0.05. Differences among dietary treatment groups were assessed using a One-Way ANOVA with Holm-Sidak post hoc test. Data sets that were not normally distributed were analyzed with a Kruskal-Wallis One-Way ANOVA on Ranks, with a Dunn's post hoc test. The response of isolated mitochondria to progressive additions of Ca^2+^ were assessed by comparing the extramitochondrial Ca^2+^ concentrations using a two way repeated measures ANOVA followed by a Holm-Sidak post hoc test for main effects and interactions.

## Supporting Information

Figure S1 **Representative spectra for mono-lyso-cardiolipin (MLCL).**
(TIF)

Figure S2 **Representative spectra for phosphotidylethanolamine (PE).**
(TIF)

Figure S3 **Representative spectra for phosphotidylcholine (PC).**
(TIF)

Figure S4 **Representative spectra for phosphotidylinositol (PI).**
(TIF)

Figure S5 **Representative spectra for phosphotidylglycerol (PG).**
(TIF)

Table S1 **Phospholipid composition of mitochondrial membranes.**
(DOCX)
