# Supplementary figures and images for: Improved Mitochondrial Function with Diet-Induced Increase in Either Docosahexaenoic Acid or Arachidonic Acid in Membrane Phospholipids
Source: PLoS One. 2012 Mar 30;7(3):e34402. doi: 10.1371/journal.pone.0034402 (PMC3316678; doi:10.1371/journal.pone.0034402)

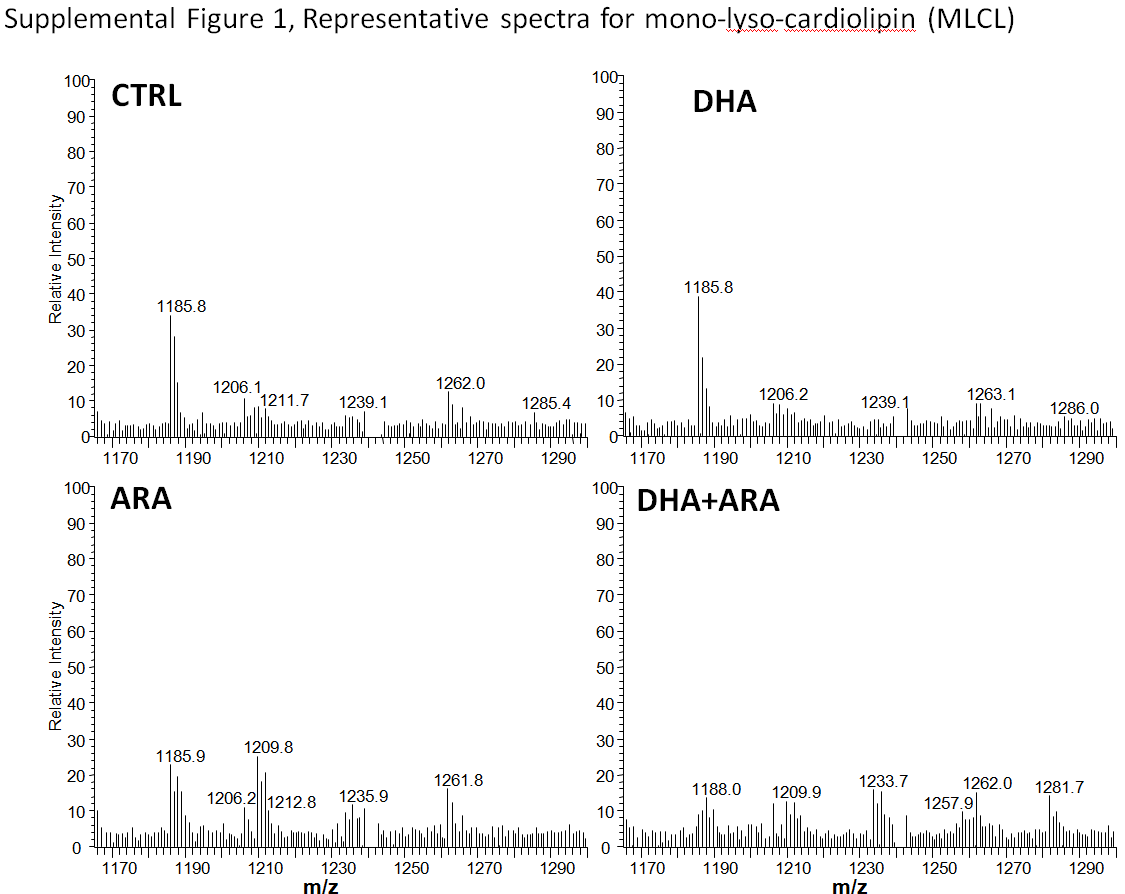

Supplement: Figure S1 — Representative spectra for mono-lyso-cardiolipin (MLCL). (TIF) [file pone.0034402.s001.tif]

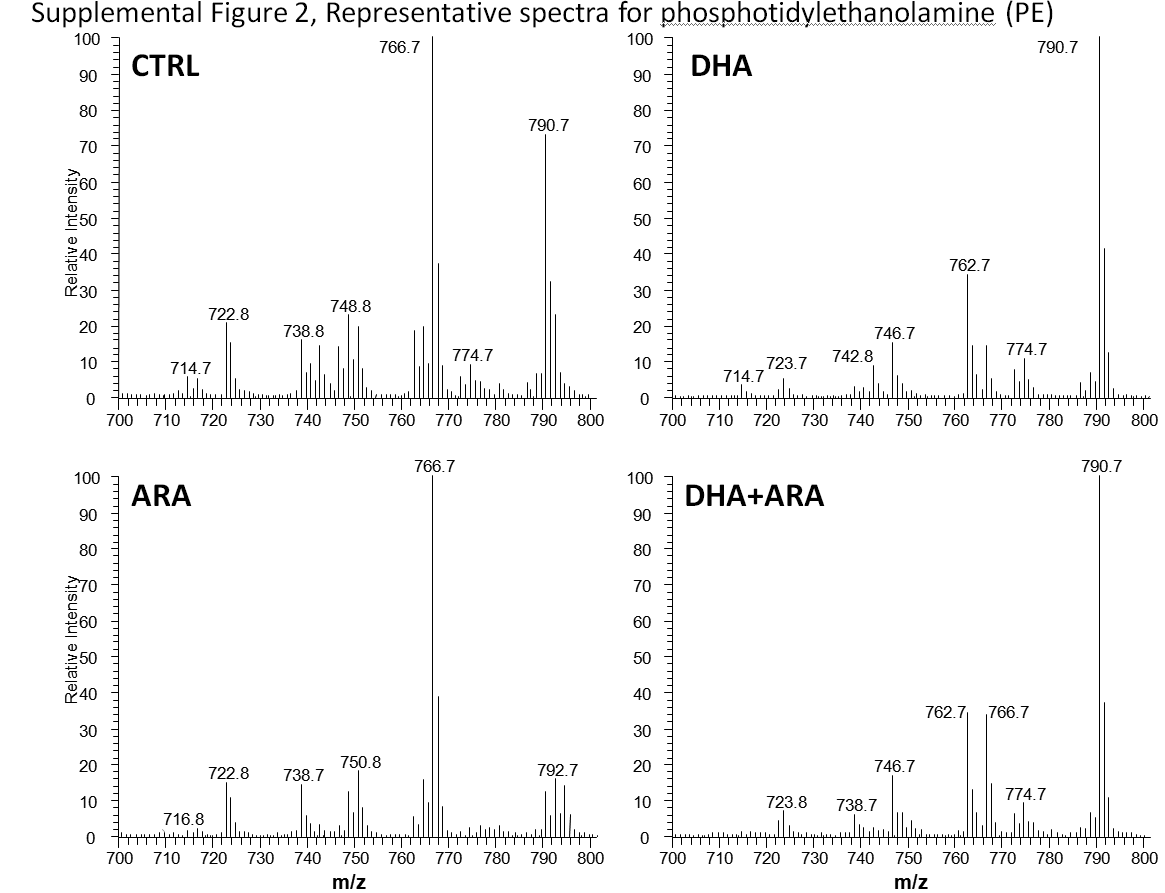

Supplement: Figure S2 — Representative spectra for phosphotidylethanolamine (PE). (TIF) [file pone.0034402.s002.tif]

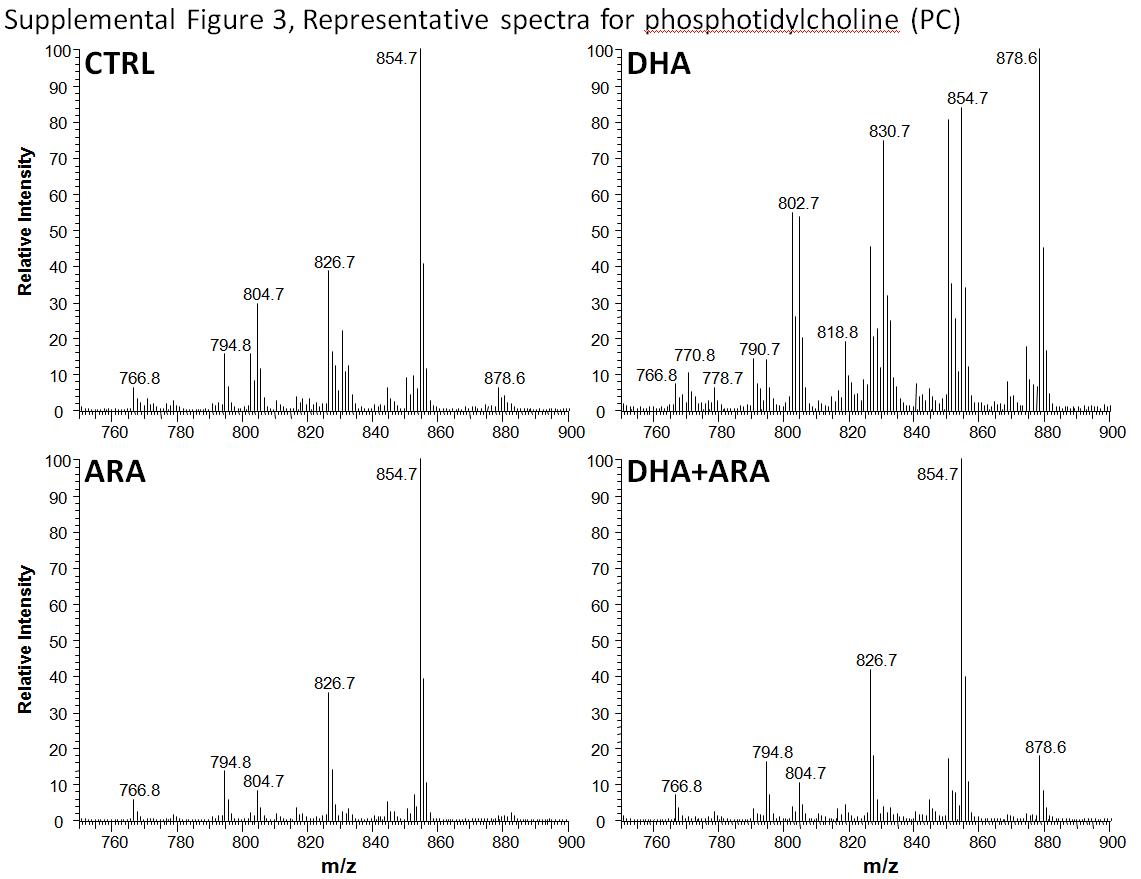

Supplement: Figure S3 — Representative spectra for phosphotidylcholine (PC). (TIF) [file pone.0034402.s003.tif]

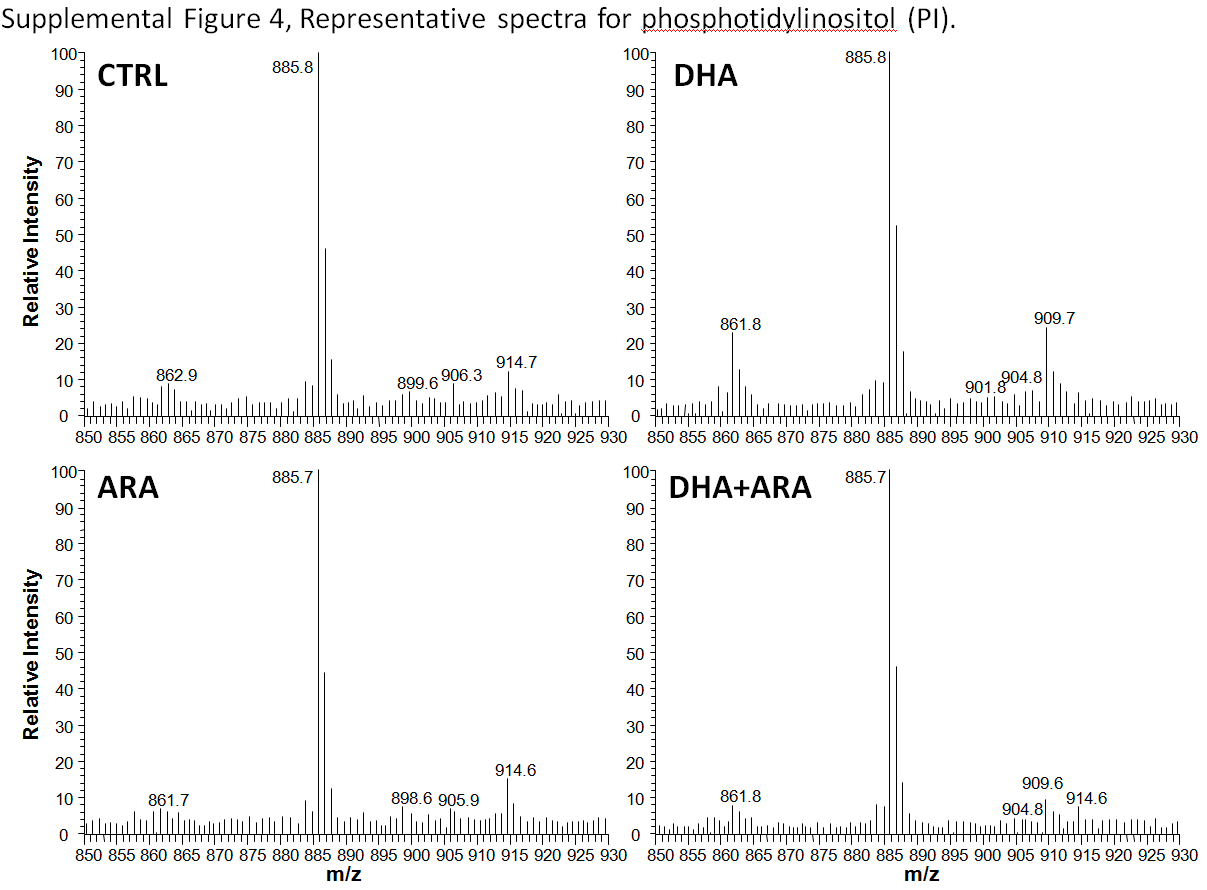

Supplement: Figure S4 — Representative spectra for phosphotidylinositol (PI). (TIF) [file pone.0034402.s004.tif]

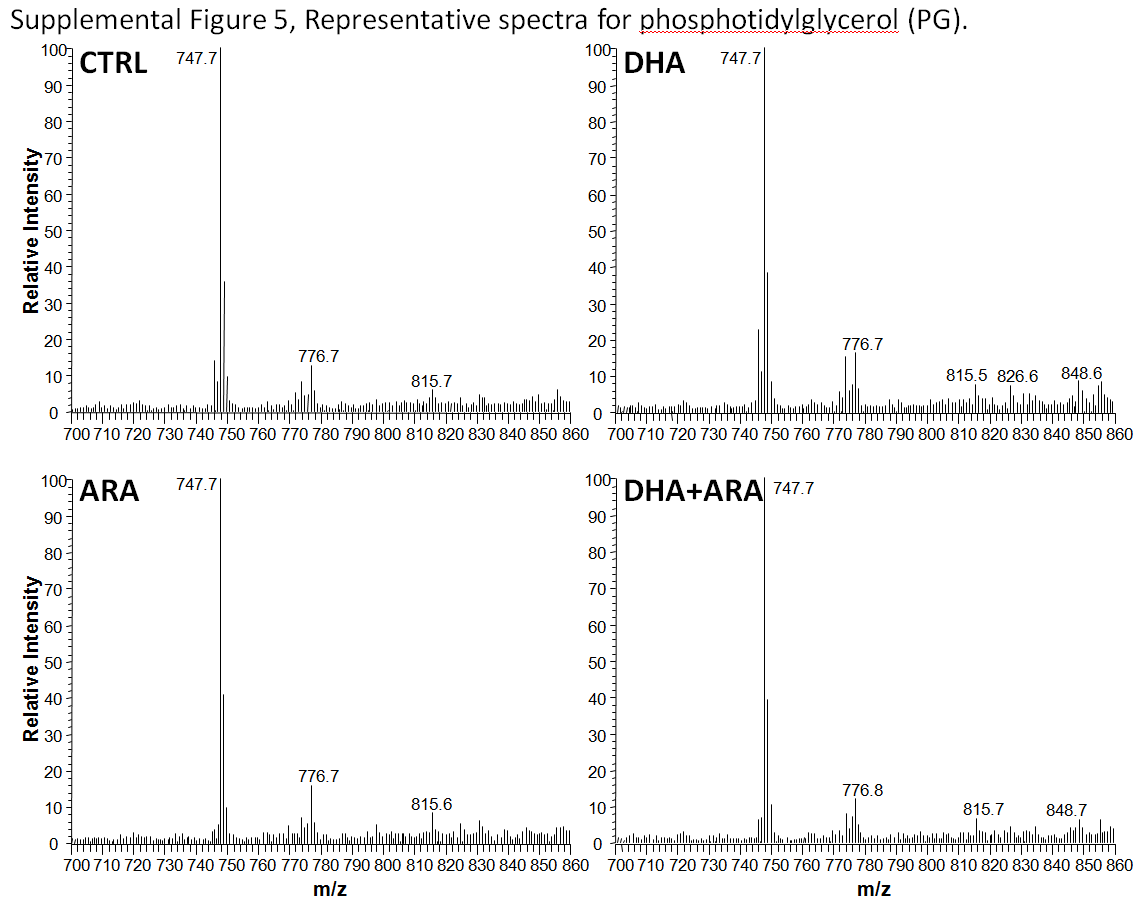

Supplement: Figure S5 — Representative spectra for phosphotidylglycerol (PG). (TIF) [file pone.0034402.s005.tif]
