# Supplementary material for: Improved Mitochondrial Function with Diet-Induced Increase in Either Docosahexaenoic Acid or Arachidonic Acid in Membrane Phospholipids
Source: PLoS One. 2012 Mar 30;7(3):e34402. doi: 10.1371/journal.pone.0034402 (PMC3316678; doi:10.1371/journal.pone.0034402)
Supplement: Table S1 — Phospholipid composition of mitochondrial membranes. (DOCX) [file pone.0034402.s006.docx]

**Supplemental Table 1. Molecular species of phospholipids in cardiac mitochondrial membranes.**

| **CL Species** | | | | **Diet** | | | |
| --- | --- | --- | --- | --- | --- | --- | --- |
| **Molecular Weight** | | | **Likely Composition** | **CTRL** | **DHA** | **ARA** | **DHA+ARA** |
| **1422.1** | **(18:2)_3_(16:1)** | | | 47.1 ± 3.2 | 26.2 ± 2.2 | 9.3 ± 0.9^*#^ | 8.5 ± 0.9^*#^ |
| **1424.1** | **(18:2)_3_(16:0) OR (16:0)_2_(18:2)(20:4)** | | | 8.5 ± 0.8 | 4.4 ± 0.8^*^ | 9.9 ± 1.0^#^ | 10.0 ± 0.9^#^ |
| **1426.1** | **(16:0)_2_(18:1)(20:4)** | | | 4.1 ± 0.4 | 4.3 ± 0.5 | 7.8 ± 0.5^*#^ | 8.5 ± 0.5^*#^ |
| **1428.1** | **(16:0)_2_(18:0)(20:4)** | | | 3.2 ± 0.2 | 2.7 ± 0.3 | 4.8 ± 0.4^*#^ | 5.6 ± 0.6^*#^ |
|  |  | | |  |  |  |  |
| **1448.1** | **(18:2)_4_** | | | 754.0 ± 60.7 | 864.8 ± 81.0 | 68.6 ± 6.1^*#^ | 46.7 ± 6.2^*#^ |
| **1450.1** | **(16:0)(18:1)(18:2)(20:4)** | | | 69.5 ± 13.6 | BQL^*^ | 67.6 ± 5.2^#^ | 49.9 ± 3.7^#^ |
| **1452.1** | **(18:2)_2_(18:1)_2_** | | | 37.2 ± 2.4 | 41.2 ± 1.3 | 40.4 ± 2.3 | 38.8 ± 2.4 |
| **1454.1** | **(16:0)(18:0)(18:1)(20:4)** | | | BQL | BQL | 5.5 ± 0.8^*#^ | 5.6 ± 0.6^*#^ |
|  |  | | |  |  |  |  |
| **1470.1** | **(16:1)(18:2)_2_(22:6)** | | | 99.7 ± 6.0 | 115.3 ± 8.6 | 20.7 ± 1.3^*#^ | 21.4 ± 1.7^*#^ |
| **1472.1** | **(18:2)_3_(20:4)** | | | 55.5 ± 5.5 | 18.0 ± 3.5^*^ | 96.9 ± 6.8^*#^ | 50.0 ± 3.5^†^ |
| **1474.1** | **(18:1)(18:2)_2_(20:4)** | | | 40.6 ± 1.7 | 38.0 ± 3.4 | 91.7 ± 5.7^*#^ | 46.3 ± 2.5^†^ |
| **1476.1** | **(18:1)_2_(18:2)(20:4)** | | | 7.0 ± 1.1 | 5.9 ± 0.7 | 29.6 ± 2.0^*#^ | 17.4 ± 1.3^*#^ |
| **1478.1** | **(18:1)_3_(20:4)** | | | 2.7 ± 0.4 | 2.9 ± 0.3 | 5.6 ± 0.6^*#^ | 5.1 ± 0.5^*#^ |
|  |  | | |  |  |  |  |
| **1496.1** | **(18:2)_3_(22:6) OR (18:2)_2_(20:4)_2_** | | | 49.1 ± 6.1 | 76.8 ± 9.1 | 83.8 ± 5.2^*^ | 80.1 ± 10.8^*^ |
| **1498.1** | **(18:1)(18:2)(20:4)_2_** | | | 21.2 ± 3.0 | 16.6 ± 6.1 | 65.2 ± 3.8^*#^ | 94.0 ± 6.9^*#^ |
| **1500.1** | **(18:1)_2_(20:4)_2_** | | | 5.6 ± 0.5 | 5.6 ± 1.5 | 37.8 ± 2.9^*#^ | 31.7 ± 4.1^*#^ |
| **1502.1** | **(18:0)(18:1)(20:4)_2_** | | | 2.4 ± 0.4 | 1.6 ± 0.3 | 15.5 ± 1.0^*#^ | 6.6 ± 0.9^*#^ |
| **1504.1** | **(18:0)_2_(20:4)_2_** | | | 1.2 ± 0.2 | 1.6 ± 0.14 | 3.2 ± 0.5^*^ | 2.0 ± 0.2 |
|  |  | | |  |  |  |  |
| **1518.1** |  | | | 7.7 ± 1.0 | 11.3 ± 1.6 | 12.0 ± 0.7 | 23.7 ± 1.4^*#†^ |
| **1520.1** | **(18:2)(20:4)_3_ OR (18:2)_2_(20:4)(22:6)** | | | 6.8 ± 1.2 | 7.8 ± 4.6 | 35.8 ± 2.6^*#^ | 68.9 ± 4.0^*#^ |
| **1522.1** | **(18:1)(20:4)_3_ OR (18:1)(18:2)(20:4)(22:6)** | | | 4.1 ± 0.4 | 5.8 ± 2.3 | 45.3 ± 3.3^*#^ | 42.9 ± 3.6^*#^ |
| **1524.1** | **(18:0)(20:4)_3_ OR (18:1)_2_(20:4)(22:6)** | | | 2.0 ± 0.4 | 1.7 ± 0.5 | 26.5 ± 2.4^*#^ | 11.3 ± 1.1^*#^ |
| **1526.1** | **(18:0)(18:1)(20:4)(22:6)** | | | 1.2 ± 0.2 | 1.4 ± 0.3 | 10.1 ± 0.7^*#^ | 4.7 ± 0.5^*†^ |
|  |  | | |  |  |  |  |
| **MLCL Species** | | | | **Diet** | | | |
| **Molecular Weight** | | **Likely Composition** | | **CTRL** | **DHA** | **ARA** | **DHA+ARA** |
| **1183.9** | | **(18:2)(16:1)(20:4)** | | 2.1 ± 0.1 | 1.8 ± 0.1 | 2.6 ± 0.2^#^ | 2.0 ± 0.1 |
| **1185.8** | | **(18:2)_3_** | | 24.0 ± 2.2 | 22.4 ± 2.8 | 8.5 ± 0.7^*#^ | 5.4 ± 0.7^*#^ |
| **1187.9** | | **(16:0)(18:1)(20:4)** | | 3.4 ± 0.6 | 2.4 ± 0.5 | 8.6 ± 0.6^*#^ | 5.7 ± 0.5^#^ |
| **1189.8** | | **(16:0)(18:0)(20:4)** | | 2.0 ± 0.2 | 1.7 ± 0.2 | 3.4 ± 0.3^*#^ | 3.3 ± 0.2^*#^ |
|  | |  | |  |  |  |  |
| **1206.1** | |  | | 4.6 ± 0.2 | 5.1 ± 0.2 | 5.0 ± 0.2 | 4.7 ± 0.2 |
| **1207.8** | | **(18:2)_2_(20:5)** | | 2.6 ± 0.4 | 3.1 ± 0.5 | 1.9 ± 0.2 | 1.9 ± 0.3 |
| **1209.9** | | **(18:2)_2_(20:4)** | | 3.6 ± 0.4 | 2.7 ± 0.3 | 12.9 ± 1.7^*#^ | 6.4 ± 0.8^#^ |
| **1211.9** | | **(18:2)(18:1)(20:4)** | | 3.1 ± 0.3 | 3.1 ± 0.2 | 7.8 ± 0.9^*#^ | 4.8 ± 0.3^#^ |
|  | |  | |  |  |  |  |
| **1261.8** | | **(18:0)(20:4)(22:6)** | | 7.8 ± 0.7 | 8.8 ± 1.0 | 10.5 ± 0.8 | 9.0 ± 0.7 |
|  | |  | |  |  |  |  |
| **PE Species** | | | | **Diet** | | | |
| **Molecular Weight** | **Likely Composition** | | | **CTRL** | **DHA** | **ARA** | **DHA+ARA** |
| **722.8** |  | | | 124.9 ± 4.4 | 28.2 ± 2.8 | 140.2 ± 6.2^*#^ | 51.0 ± 2.7^*#^ |
| **724.8** |  | | | 20.8 ± 1.1 | 24.5 ± 1.1 | 20.8 ± 1.2 | 22.1 ± 0.8 |
|  |  | | |  |  |  |  |
| **736.7** | **(16:0)(20:5)** | | | 9.4 ± 1.1 | 10.6 ± 1.0 | 11.5 ± 0.7 | 8.7 ± 0.5 |
| **738.8** | **(16:0)(20:4)** | | | 102.0 ± 5.1 | 35.1 ± 3.0 | 106.8 ± 6.1^*#^ | 60.0 ± 3.8^*#^ |
| **740.7** | **(18:1)(18:2)** | | | 36.0 ± 3.9 | 25.3 ± 1.5 | 15.4 ± 1.0^*#^ | 13.7 ± 1.0^*#^ |
| **742.8** | **(18:1)_2_** | | | 76.7 ± 7.4 | 87.1 ± 7.8 | 20.2 ± 1.1^*#^ | 18.4 ± 1.2^*#^ |
| **744.8** | **(18:0)(18:1)** | | | 15.7 ± 1.1 | 8.0 ± 0.9^*^ | 13.4 ± 1.0^#^ | 13.8 ± 0.5^#^ |
| **746.7** | **(18:0)_2_** | | | 106.3 ± 7.4 | 183.9 ± 14.9^*^ | 29.0 ± 1.4^#^ | 178.1 ± 9.5^*†^ |
| **748.8** |  | | | 124.6 ± 5.0 | 24.3 ± 2.2^*^ | 126.4 ± 5.3^#^ | 38.3 ± 1.7^*†^ |
| **750.7** |  | | | 104.8 ± 5.1 | 21.9 ± 2.9^*^ | 191.4 ± 12.8^#^ | 39.1 ± 1.8^†^ |
| **752.7** |  | | | 6.6 ± 0.5 | 6.1 ± 0.8 | 9.9 ± 1.1 | 6.8 ± 0.7 |
|  |  | | |  |  |  |  |
| **760.7** | **(16:1)(22:6)** | | | 7.8 ± 0.7 | 13.2 ± 1.1^*^ | 4.9 ± 0.4^*#^ | 12.5 ± 0.8^*†^ |
| **762.7** | **(16:0)(22:6)** | | | 178.5 ± 10.8 | 383.8 ± 15.4^*^ | 48.6 ± 1.9^#^ | 351.6 ± 18.3^*†^ |
| **764.7** | **(18:1)(20:4)** | | | 102.1 ± 5.2 | 24.9 ± 2.0^*^ | 137.0 ± 6.0^#^ | 21.9 ± 1.7^*†^ |
| **766.8** | **(18:0)(20:4)** | | | 603.5 ± 23.2 | 161.1 ± 14.6^*^ | 893.3 ± 43.1^#^ | 340.3 ± 16.2^†^ |
| **768.8** |  | | | BQL | 1.5 ± 1.7^*^ | BQL^#^ | BQL^#^ |
|  |  | | |  |  |  |  |
| **788.7** | **(18:1)(22:6)** | | | 57.2 ± 2.5 | 82.0 ± 4.2^*^ | 14.9 ± 1.6^*#^ | 76.0 ± 3.6^†^ |
| **790.7** | **(18:0)(22:6)** | | | 592.4 ± 34.6 | 1139.2 ± 48.6^*^ | 149.6 ± 7.5^#^ | 1015.7 ± 55.1^*†^ |
| **792.7** |  | | | 79.3 ± 8.9 | BQL^*^ | 207.4 ± 12.2^#^ | BQL^*†^ |
| **794.6** |  | | | 17.9 ± 1.6 | 12.5 ± 1.0 | 148.3 ± 8.9^*#^ | 14.2 ± 0.8^†^ |
| **796.3** |  | | | 7.8 ± 0.7 | 5.0 ± 0.3 | BQL^*#^ | 5.1 ± 0.4^†^ |
|  |  | | |  |  |  |  |
| **PI Species** | | | | **Diet** | | | |
| **Molecular Weight** | **Likely Composition** | | | **CTRL** | **DHA** | **ARA** | **DHA+ARA** |
| **861.6** |  | | | 4.4 ± 0.6 | 5.8 ± 0.3^*^ | 5.0 ± 0.3 | 4.4 ± 0.3^#^ |
| **863.5** |  | | | 8.4 ± 0.4 | 10.4 ± 0.8 | 6.3 ± 0.6^*#^ | 7.2 ± 0.3^#^ |
| **865.0** |  | | | 3.6 ± 0.3 | 3.5 ± 0.2 | 4.0 ± 0.2 | 3.3 ± 0.2 |
|  |  | | |  |  |  |  |
| **883.7** | **(18:1)(20:5)** | | | 12.0 ± 1.0 | 10.8 ± 0.9 | 13.2 ± 1.2 | 10.2 ± 0.6 |
| **885.7** | **(18:0)(20:4)** | | | 157.7 ± 10.4 | 112.9 ± 8.5 | 148.2 ± 13.5 | 151.8 ± 11.9 |
| **887.7** | **(18:0)(20:3)** | | | BQL | 1.2 ± 0.6^*^ | BQL^#^ | BQL^#^ |
|  |  | | |  |  |  |  |
| **906.3** | **(18:2)(22:6)** | | | 6.5 ± 0.5 | 7.0 ± 0.3 | 6.8 ± 0.4 | 6.4 ± 0.5 |
| **908.3** | **(18:1)(22:6)** | | | 1.7 ± 0.4 | 3.1 ± 0.4 | 2.2 ± 0.3 | 1.7 ± 0.5 |
| **909.7** | **(18:0)(22:6)** | | | 5.3 ± 0.4 | 23.7 ± 3.0^*^ | 3.7 ± 0.3^#^ | 13.7 ± 1.4^*†^ |
| **911.6** |  | | | 5.1 ± 0.5 | 5.0 ± 0.5 | 4.1 ± 0.3 | 4.5 ± 0.2 |
| **912.6** |  | | | 4.7 ± 0.3 | 4.9 ± 0.4 | 4.1 ± 0.3 | 3.6 ± 0.2^#^ |
| **914.4** |  | | | 12.6 ± 1.0 | 5.1 ± 0.5^*^ | 17.8 ± 1.1^#^ | 8.4 ± 0.8^†^ |
| **916.8** |  | | | 3.0 ± 0.3 | 2.9 ± 0.2 | 2.2 ± 0.3 | 2.8 ± 0.1 |
| **918.8** |  | | | 3.0 ± 0.3 | 2.6 ± 0.2 | 2.8 ± 0.2 | 3.0 ± 0.3 |
|  |  | | |  |  |  |  |
| **PG Species** | | | | **Diet** | | | |
| **Molecular Weight** | **Likely Composition** | | | **CTRL** | **DHA** | **ARA** | **DHA+ARA** |
| **745.7** | **(16:0)(18:2)** | | | 32.8 ± 2.4 | 39.5 ± 2.9 | 8.8± 1.1^*#^ | 11.3 ± 1.1^*#^ |
| **747.7** | **(16:0)(18:1)** | | | 242.2 ± 9.7 | 206.0 ± 8.9 | 251.2 ± 13.6^#^ | 252.2 ± 12.0^#^ |
|  |  | | |  |  |  |  |
| **769.8** | **(16:0)(20:4)** | | | 5.6 ± 0.4 | 5.0 ± 0.3 | 10.8 ± 0.8^*#^ | 6.6 ± 0.4^†^ |
| **771.6** | **(18:1)(18:2)** | | | 10.5 ± 0.7 | 8.8 ± 0.6 | 5.4 ± 0.5^*#^ | 6.6 ± 0.4^*#^ |
| **773.7** | **(18:1)_2_** | | | 22.0 ± 1.1 | 25.9 ± 1.7 | 17.4 ± 1.1^#^ | 15.2 ± 0.8^*#^ |
| **775.7** | **(18:0)(18:1)** | | | 12.1 ± 1.0 | 11.1 ± 0.5 | 15.4 ± 1.3^#^ | 11.9 ± 0.8 |
| **776.7** | **(18:0)(18:0)** | | | 28.2 ± 0.7 | 28.9 ± 0.7 | 29.4 ± 0.8 | 28.3 ± 1.9 |
|  |  | | |  |  |  |  |
| **PC Species** | | | | **Diet** | | | |
| **Molecular Weight** | **Likely Composition** | | | **CTRL** | **DHA** | **ARA** | **DHA+ARA** |
| **790.7** |  | | | 10.7 ± 0.5 | 30.8 ± 2.5* | 4.6 ± 0.2^#^ | 22.0 ± 1.4^†^ |
|  |  | | |  |  |  |  |
| **802.7** | **(16:0)(18:2)** | | | 54.6 ± 4.5 | 108.1 ± 5.9 | 11.6 ± 0.7*^#^ | 15.5 ± 1.0*^#^ |
| **804.7** | **(16:0)(18:1)** | | | 112.2 ± 4.6 | 86.4 ± 4.6* | 42.7 ± 2.4*^#^ | 60.2 ± 3.6*^#†^ |
| **806.7** | **(16:0)(18:0)** | | | BQL | 0.6 ± 0.2* | 2.4 ± 0.2*^#^ | 1.7 ± 0.2*^#†^ |
|  |  | | |  |  |  |  |
| **810.7** |  | | | 14.7 ± 1.1 | 6.0 ± 0.4* | 13.3 ± 0.9^#^ | 13.3 ± 0.7^#^ |
| **812.7** |  | | | 1.7 ± 0.1 | 1.5 ± 0.1 | 2.3 ± 0.1*^#^ | 2.7 ± 0.1*^#†^ |
| **814.7** |  | | | 1.9 ± 0.1 | 6.6 ± 0.2* | 1.3 ± 0.1^#^ | 2.2 ± 0.1^†^ |
| **816.7** |  | | | 6.8 ± 1.3 | 5.6 ± 0.5 | 7.8 ± 1.6 | 8.6 ± 1.24 |
| **818.7** |  | | | 9.4 ± 0.4 | 37.8 ± 2.9* | 4.3 ± 0.4^#^ | 20.3 ± 1.4^†^ |
|  |  | | |  |  |  |  |
| **826.7** | **(16:0)(20:4)** | | | 214.6 ± 11.0 | 102.6 ± 5.9* | 244.4 ± 13.0^#^ | 277.3 ± 11.6*^#^ |
| **828.7** | **(18:1)(18:2)** | | | 24.7 ± 3.1 | 30.6 ± 2.0 | BQL*^#^ | BQL*^#^ |
| **830.7** | **(18:0)(18:2) OR (18:1)_2_** | | | 81.6 ± 72.0 | 149.4 ± 8.1 | 12.9 ± 0.8*^#^ | 13.5 ± 0.9*^#^ |
| **832.7** | **(18:0)(18:1)** | | | 43.4 ± 1.8 | 31.1 ± 1.5 | 17.2 ± 0.7*^#^ | 17.5 ± 0.8*^#^ |
| **834.7** | **(18:0)_2_** | | | 1.5 ± 0.2 | 9.7 ± 0.9* | 1.2 ± 0.1^#^ | 7.3 ± 0.4*^†^ |
|  |  | | |  |  |  |  |
| **840.6** |  | | | 4.6 ± 0.3 | 6.5 ± 1.2 | 6.5 ± 0.4 | 9.8 ± 0.6*^#^ |
| **842.6** |  | | | 3.2 ± 0.3 | 3.2 ± 0.6 | 2.9 ± 0.3 | 3.5 ± 0.4 |
| **844.7** |  | | | 26.5 ± 3.7 | 9.2 ± 1.3* | 31.9 ± 3.8^#^ | 31.8 ± 6.3^#^ |
| **846.7** |  | | | 7.5 ± 1.0 | 3.7 ± 0.4 | 8.7 ± 0.9^#^ | 8.7 ± 1.4^#^ |
|  |  | | |  |  |  |  |
| **850.7** | **(16:0)(22:6)** | | | 51.7 ± 2.5 | 172.8 ± 13.8* | 17.5 ± 1.0^#^ | 115.5 ± 6.2^†^ |
| **852.7** | **(16:0)(20:3)** | | | 43.1 ± 1.7 | 26.6 ± 2.3* | 45.1 ± 2.3^#^ | 28.1 ± 1.1*^†^ |
| **854.7** | **(18:0)(20:4)** | | | 527.1 ± 28.1 | 196.51 ± 8.3* | 827.8 ± 49.5*^#^ | 554.2 ± 26.6^#†^ |
|  |  | | |  |  |  |  |
| **874.6** | **(20:4)_2_ OR (18:2)(22:6)** | | | 5.6 ± 0.5 | 32.9 ± 1.5* | 3.3 ± 0.2^#^ | 6.5 ± 0.8^#^ |
| **876.6** | **(18:1)(22:6)** | | | 3.4 ± 0.3 | 8.5 ± 0.5* | 1.6 ± 0.1^#^ | 4.4 ± 0.6^#†^ |
| **878.6** | **(18:0)(22:6)** | | | 29.2 ± 3.7 | 201.0 ± 14.9* | 6.8 ± 0.3^#^ | 74.4 ± 12.7^#†^ |

CL, cardiolipin. MLCL, monolysocardiolipin. PE, phosphotidylethanolamine. PI, phosphotidylinositol. PG, phosphotidylglycine. PC, phosphotidylcholine. BQL, Below Quantifiable Limit. Data are presented as mean peak intensity in ten thousand arbitrary units. Data are mean±SEM. CTRL, n=14. DHA, n=13. ARA, n=13. DHA+ARA, n=14. *p<0.05 vs CTRL. #p<0.05 vs DHA, †p<0.05 vs ARA.
